# Whole-genome sequencing of three extremophile *Bacillus* sp. strains isolated from the Atacama Desert

**DOI:** 10.1128/mra.00679-24

**Published:** 2024-12-16

**Authors:** Nicole T. Cavanaugh, Girish Kumar, Matteo Couto Frignani, Nathan Thewedros, Marcello Twahirwa, Carlos Riquelme, André O. Hudson, Yunrong Chai, Veronica Godoy-Carter

**Affiliations:** 1 Department of Biology, Northeastern University College of Science, Boston, Massachusetts, USA; 2 Thomas H. Gosnell School of Life Sciences, Rochester Institute of Technology College of Science, Rochester, New York, USA; 3 Instituto de Bioinnovacion, Universidad de Antofagasta, Antofagasta, Chile; The University of Arizona, Tucson, Arizona, USA

**Keywords:** extremophiles, environmental microbiology, biofilms, genomes, genomics

## Abstract

The Atacama Desert is home to bacteria that use biofilms as a means of protecting themselves against the harsh environment. To inform research regarding this survival mechanism, we cultured and sequenced the genomes of three *Bacillus* sp. isolates from Atacama Desert soil.

## ANNOUNCEMENT

The Atacama Desert is one of the most ancient, most highly irradiated, and driest deserts in the world (1). Despite this, it is home to a variety of bacterial life. Bacteria in this region are shown to have developed adaptations to survive this harsh, irradiated environment such as high levels of pigment production and biofilm formation (2). In the past, most research addressing survival mechanisms in microbes from the Atacama have focused on archaea. We are studying how eubacteria have developed adaptations to survive in such a desert environment.

**Fig 1:**
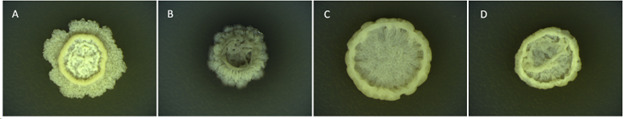
Environmental strains isolated from the Atacama Desert form colony biofilms. Colony biofilms of 0102A (**A**), 0209A (**B**), 0909A (**C**), and 3610 (12), (**D**) grown on LBGM medium (11) after 48 h of incubation.

To study how biofilms impact bacterial survival, we started by culturing and isolating a variety of bacterial colonies from desert soil and screening for biofilm-forming isolates. Bacterial colonies were purified after plating 100 µL of a soil water slurry on Reasoner’s 2A (R2A) agar medium and incubating plates at 28°C for 48–72 h. To identify the isolates, we performed 16S rRNA gene sequencing on the V3/4 variable regions using the following primers: 5′-CCTACGGGNGGCWGCAG-3′ and 5′-GACTACHVGGGTATCTAATCC-3′. Using National Society for Biotechnology Information (NCBI) BLASTN version 2.15.0, three *Bacillus* sp. were identified (3, 4). The *Bacillus* genus contains Gram-positive bacteria that are found throughout the environment, many of which are known to produce biofilms (5–10). To test biofilm formation, strains were grown at 37°C to OD600 = 1.0 in Luria–Bertani (LB) broth, and 2 µL of culture was spotted on LB agar plates supplemented with 1% glycerol and 0.1 mM MnSO4 (LBGM, biofilm-inducing medium) (11). Colony biofilms were incubated for 48 h (Fig. 1).

These *Bacillus* sp. were selected for further genomic analysis. Genomic DNA was isolated from 2-mL LB cultures prepared from single colonies using the Wizard Genomic DNA Purification Kit (Promega). DNA libraries were prepared using the Nextera XT library preparation kit (Illumina). Libraries were sequenced using the Illumina MiSeq System using the MiSeq Reagent V3 kit (2 × 300 cycles) (Illumina) at the Genomics Lab, Rochester Institute of Technology. Raw reads were trimmed using Trimmomatic Galaxy version 0.38.1 (13). The quality of the trimmed reads was assessed using FastQC version 0.74+galaxy0 (14). Trimmed reads over 100 bp were assembled *de novo* using Unicycler version 0.5.0+galaxy1 (15). Quality analysis was performed using Quast version 5.2.0+galaxy1 (16). Quality assessments are outlined in Table 1. All programs used were run at default parameters unless otherwise noted. Analysis was performed using the Prokaryotic Genome Analysis Pipeline (PGAP) version 6.6 to reveal the read length and total number of genes, protein coding sequences, rRNAs, and tRNAs in each assembly (Table 1) (17–19). PGAP filters out reads under 200 bp before assembly and analysis. Further analysis of the *eps* operon in each genome using NCBI BLASTN version 2.14.1+galaxy and taxonomy analysis by Automated Multi Locus Species Tree (autoMLST) showed that these three isolates were most closely related to *Bacillus vallismortis and Bacillus mojavensis* (3, 4, 20).

**TABLE 1:** Summary of quality assessments for each strain^*a*^

Isolate	Geographic locations	Elevation (ft)	Most closely related species	Total bases (Mbp)	Assembly size (bp)	Number of reads (FastQC)	Read length	Number of contigs	N50 (bp)	GC content	Total genes	Protein coding sequences	rRNA operons	tRNAs
0102A	22.56.56 S 68.18.53 W	8,340	*B. vallismortis*	813.2	4,192,438	4,406,089	2 × 289	27	350,970	43.87%	4,296	4,067	3	76
0209A	22.31.54 S 68.2.27 W	14,000	*B. vallismortis*	980.4	4,167,904	5,239,849	2 × 285	19	1,048,338	43.98%	4,208	3,989	3	73
0909A	22.31.54 S 68.2.27 W	14,000	*B. mojavensis*	857.1	3,899,559	4,586,376	2 × 286	19	930,776	43.66%	4,061	3,870	3	67

^*a*^ A summary of the identities and quality assessments of each strain 0102A, 0209A, and 0909A. The latitude and longitude and elevation, where each sample was collected, are summarized. For each strain, the total bases and number of assembly size were determined using FastQC Galaxy version 0.74+galaxy0. Read length, number of contigs, N50, and GC content were assessed using Quast Galaxy version 5.2.0+galaxy1. Read length and total genes, protein coding sequences, rRNAs, and tRNAs were determined using the Prokaryotic Genome Analysis Pipeline (PGAP) version 6.6.

## Data Availability

The WGS projects for Bacillus subtilis strains 0102A, 0209A, and 0909A have been deposited in GenBank under BioProject number PRJNA1071648. Strain 0102A is deposited under BioSample number SAMN39706723 and SRA number SRR27839954. PGAP analysis can be found under accession number JBAIZW000000000. Strain 0209A is deposited under BioSample number SAMN39706724 and SRA number SRR27839953. PGAP analysis can be found under accession number JBAIZV000000000. Strain 0909A is deposited under BioSample number SAMN39706725 and SRA number SRR27839952. PGAP analysis can be found under accession number JBAIZU000000000. The NCBI BLASTN webserver was used to identify the 0102A, 0209A, and 0909A 16 s rRNA gene sequence. All programs found on the Galaxy platform can be found at usegalaxy.org.
